# An open-access and inexpensive 3D printed otoscope for low-resource settings and health crises

**DOI:** 10.1186/s41205-021-00127-3

**Published:** 2021-11-17

**Authors:** Matteo Capobussi, Lorenzo Moja

**Affiliations:** 1 Department of Biomedical Sciences for Health, University of Milan, Milan, Italy; 2 Interuniversity Center in Clinical Research, University of Milan, Milan, Italy

**Keywords:** Maker Culture, Family Medicine, Otoscope, Prototyping, 3D printing

## Abstract

Limited access to key diagnostic tools is detrimental to priority health needs of populations. Ear pain, tenderness, itching, and different degree of hearing loss are common problems which require otoscopy as first diagnostic assessment. Where an otoscope is not available because of budget constraints, a self-fabricated low-cost otoscope might represent a feasible opportunity. In this paper, we share the design and construction process of an open-source, 3D printed, otoscope. The prototype was compared to a commercial solution, demonstrating similar overall quality between the instruments.

**Supplementary Information:**

The online version contains supplementary material available at 10.1186/s41205-021-00127-3.

## Introduction

The last few years saw an increased availability of low-cost 3D printers and their growing diffusion among academic and hobbyist communities [1]. In medicine, these developers are capable of taking advantage of innovative technologies, including 3D printing [2, 3], micro-soldering, and electronic circuit design, to create assistive technologies that improve clinical practice or increment cost-effectiveness. The ability to progress “open” projects for free, and the ease in product customization, determined the increasing success of fabrication laboratories in medicine, especially for prosthetics [4, 5], splints, [6] and preoperative planning [7]. These creative spaces are now widespread and hold the capability of starting small-scale productions [8]. These features proved vital during recent health crises, such as the Nepal earthquake of 2015 or the COVID-19 pandemic [9–12]. In both these situations, Maker-designed devices were able to provide help to the populations in need. However, their impact was limited. Existing devices had to be reverse-engineered by consumer-maker communities and tested on the field, before local fabrication laboratories could begin. Therefore, open access medical device projects should be already available and validated before a new crisis arises [13]. The purpose of this article is to provide a description of a 3D printed, affordable, yet meeting high- quality standards, otoscope. The costs of medical-grade, commercially available otoscopes remain high, constituting a barrier for an adequate hearing healthcare in most low-resources medical settings globally [14]. Here we share our design and construction process as an open-source project realized through inexpensive 3D printing, hoping others can replicate and validate our prototype, where commercial solutions are demanding.

## Methods

The otoscope modern forms have undergone little evolution since their invention [15]. The technologies involved are easy to replicate using commonly available electronics and magnifying systems, making them ideal targets for Maker’s reverse-engineering.

### Early prototype design and its disadvantages

**Fig. 1:**
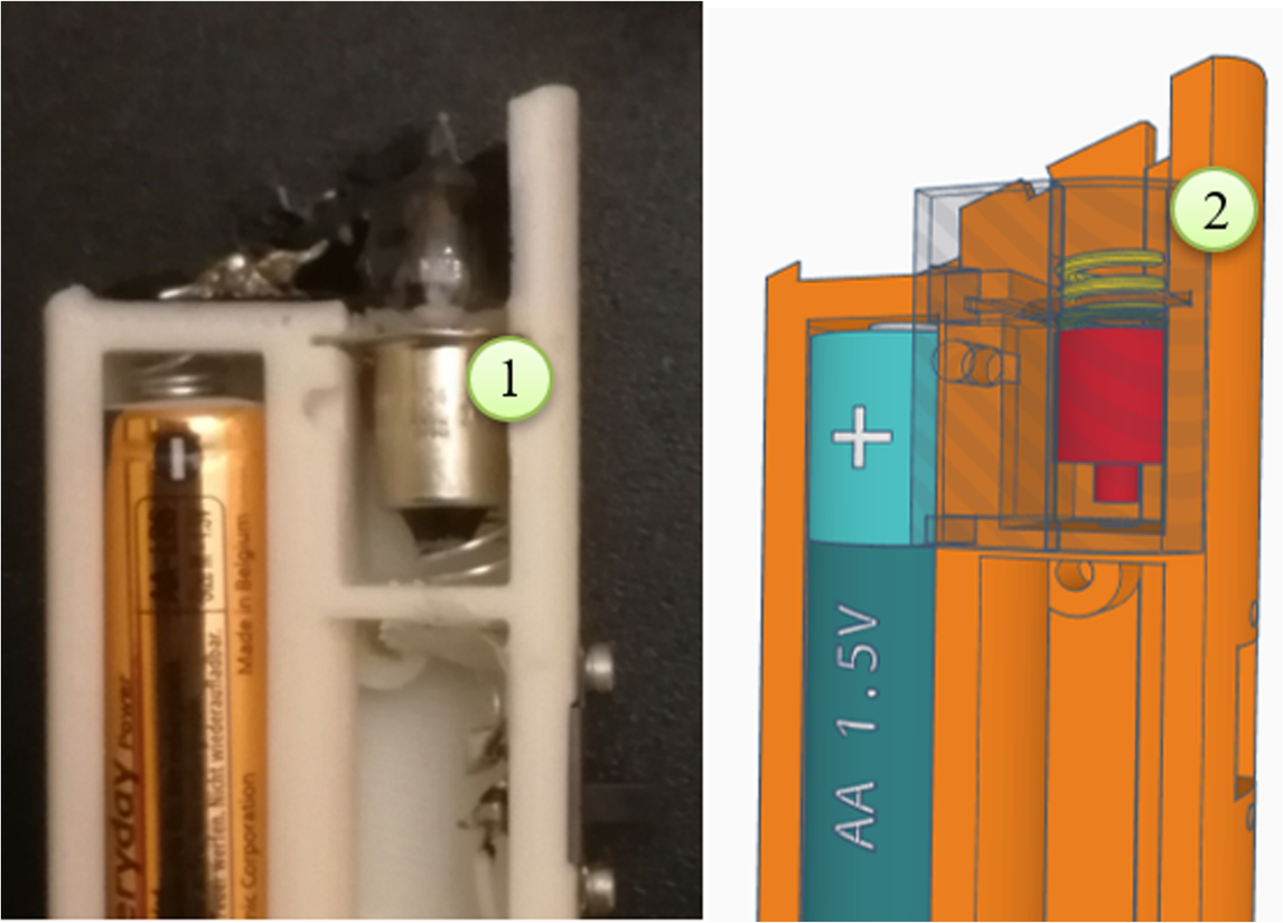
The first prototype, inspired by currently available otoscopes, included a housing for a lightning bulb (1) and a small door (2) for replacing it when needed

Our first prototype, inspired by otoscopes currently available for sale, included an ABS shell enclosing an AA battery compartment, a small incandescence light bulb, a magnifying lens and an optic-fiber light distribution system (Fig. 1). However, this approach presented several limitations. Some components, i.e., the optic fibers, are not easily available in the market and may require long times for delivery. For these reasons are not commonly used by Makers, preferring LED light sources. Another issue was the brightness of the light bulb we used, that proved to be insufficient when light had to cross “transparent” 3D printed resin. Consequently, we abandoned this design.

### Modular advanced prototype design

**Fig. 2:**
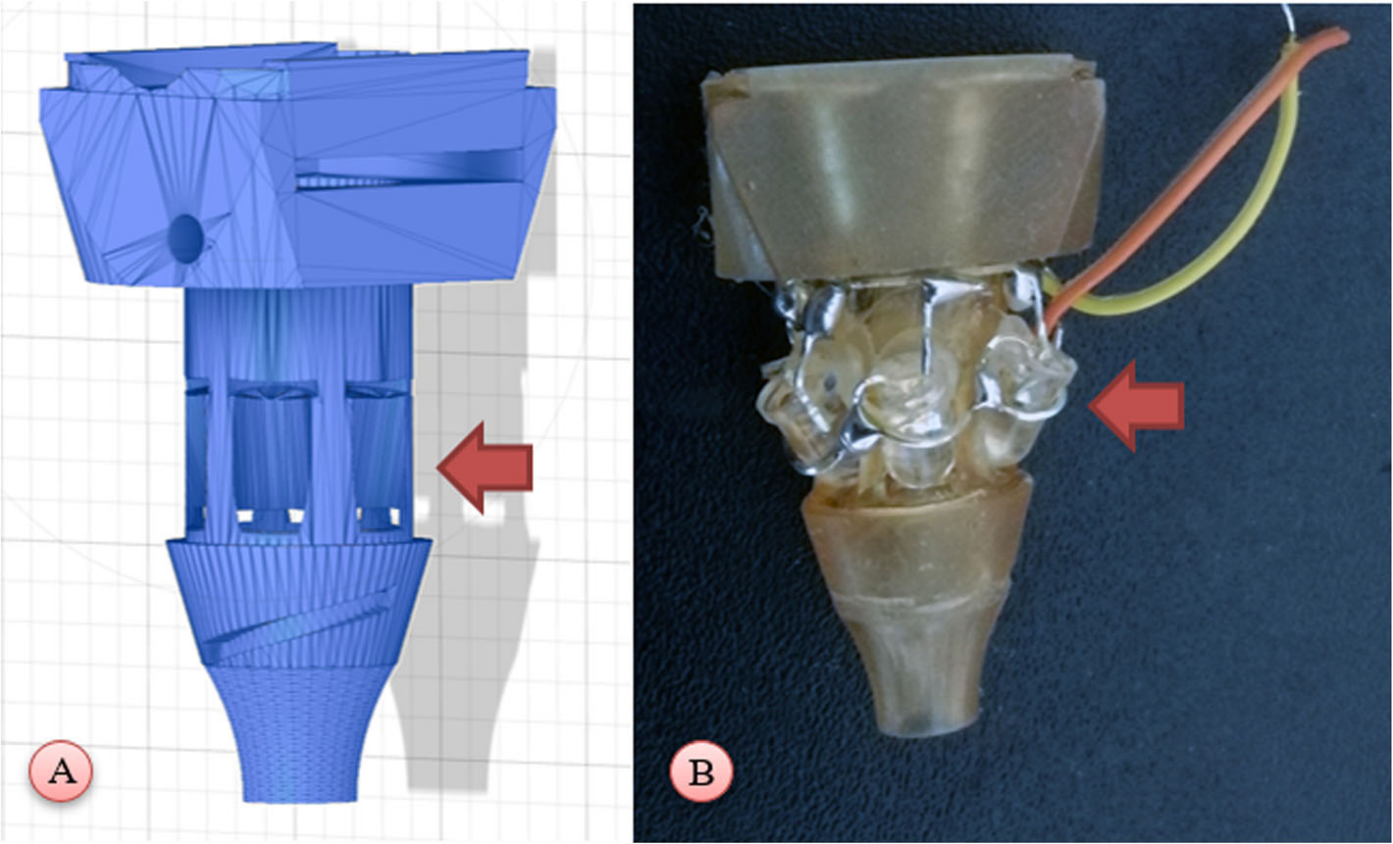
3D representation (**A**) of the adopted LED housing and picture (**B**) of the lightning system (red arrows)

We decided to completely redesign the device starting from items normally available. Through trial and error, we tested and then adopted a six 5mm, 3.3 V, white, LEDs lightning system, which empirically seemed to provide the same amount of light of the commercial solution. We accommodated the LEDs in a ring shape around the visual pathway of the user and placed them as close to the target as possible, to minimize the loss of light through resin (Fig. 2). Then, we designed a 3D printed lens system: SLA printers have a sufficient resolution to print an entire optical block with acceptable results [16]. However, the post processing steps needed to produce quality lenses are time consuming, and require optical clear, expensive resin, limiting feasibility. For these reasons we privileged as pragmatic solution the use of a magnification system based on industrial Fresnel lenses. These are inexpensive and can be easily acquired on e-commerce websites. We chose a 3x magnification, credit card sized, lens as it is one of the most common available lenses for purchase. In order to allow foreign body extraction procedures, we designed a removable frame for the lenses. Otopneumoscopy is also possible through a small hole designed for positive pressure tubes. Though, it should be noted that FDM prints are not airtight. If this use is anticipated, we recommend printing the whole head with SLA technology. We did not use biocompatible resin since the head is never in contact with patient’s skin, as disposable cones act as separators.

We envisaged that we could decrease costs by adopting a modular approach: designing a multi-use handle, which contains the batteries and alimentation system, compatible with multiple heads. These could be switched, transforming the device in an otoscope, a dermatoscope, or even an ophthalmoscope, depending on needs. Looking through materials already at our disposal, we chose a nickel plating for facilitating the head change procedure.

All 3D designs were performed using TinkerCAD (www.tinkercad.com, Autodesk Inc., San Rafael, California, USA). Then we manufactured the items using a Prusa i3 Pro-B (Geeetech Ltd, Shenzhen, China) fusion deposition modeling (FDM) 3D printer, and a Mars Pro (Elegoo Inc., Shenzhen, China) stereolithography (SLA) printer. All these technologies are open source.

For FDM, we used ABS filament extruded at 240 °C with a heated bed (100 °C), running at 50 mm/s with a layer height resolution of 0.2mm. Pieces were oriented mostly on the horizontal plane to minimize the need for supports. For SLA, we used standard Elegoo transparent resin with default parameters, 7 s layer cure time, 150mm/min retract speed and 0.05 mm resolution. We used a 30° orientation and a careful positioning of supports to minimize failures caused by suction effect. All .stl files, already in the correct orientation, are provided in supplementary material.

**Fig. 3:**
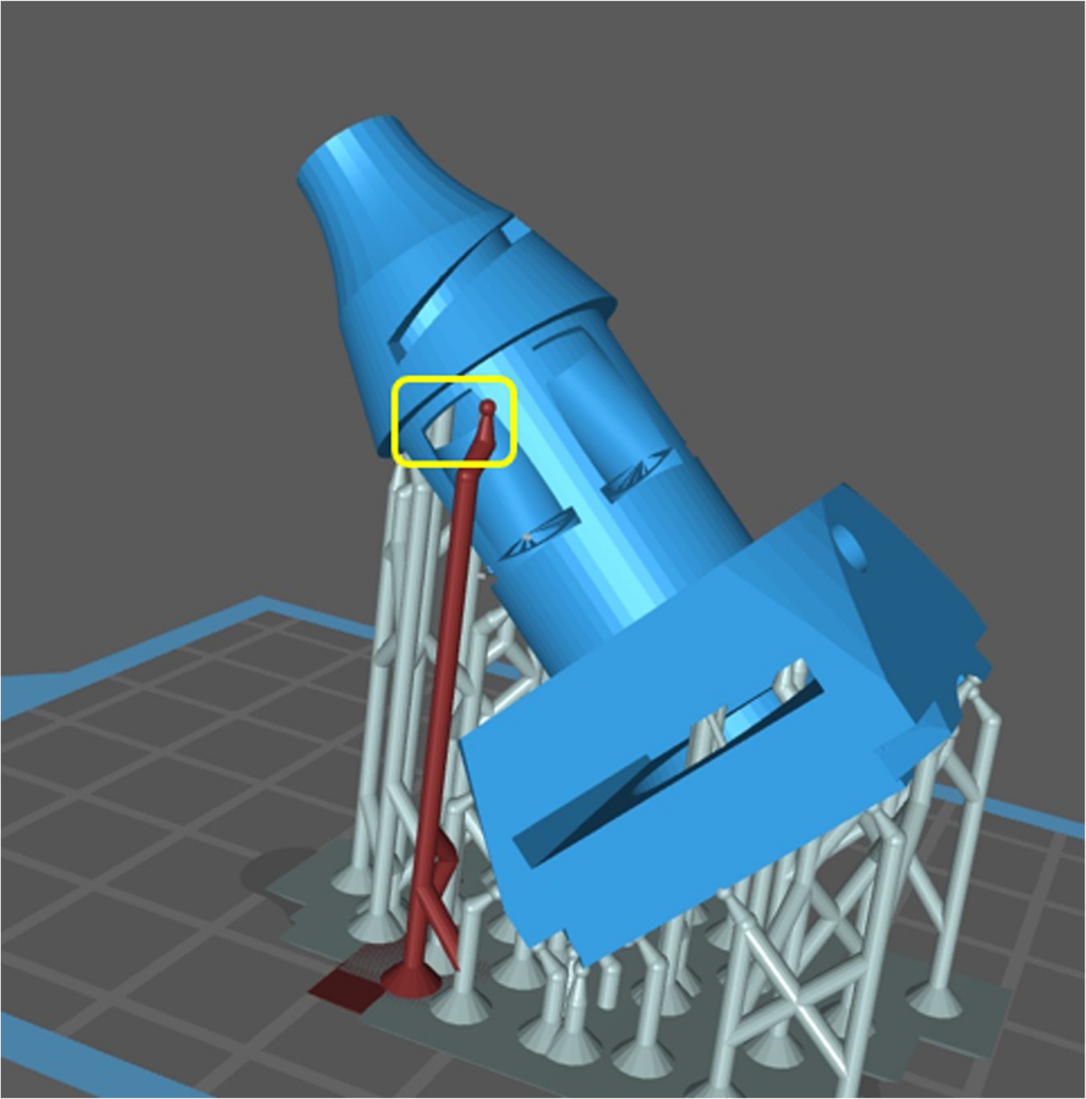
Removing the diaphragm in front of the UV LEDs (yellow) may cause print failures. To avoid this problem, we recommend supplementary supports (red)

Then, we designed a second head lodging UV LEDs. Wood’s lamp is used in dermatology to diagnose fungal infections. Differential diagnosis between fungal and bacterial external otitis poses important challenges to clinicians by sharing common signs. We hypothesized that many fungal infections of the ear canal, as well as some bacterial infections, could show the specific fluorescence patterns recognized during dermatologic examinations [17, 18]. As fluorescence requires direct illumination from short wavelength light, resin diaphragms between the LEDs and their targets were removed. The resulting prototype was able to elicit fluorescence on test materials (e.g., UV polymerizing resin for 3D printing, industrial soap). Printing the emptied shell resulted in a slightly higher print failure rate, however imperfections in prints could be easily corrected by careful use of a glue gun during LED positioning (Fig. 3).

Finally, we compared the technical features of our self-fabricated white- and UV-light 3D printed otoscopes with a commercially available otoscope (Sigma F.O. LED, G.I.M.A. S.p.A., Milan, Italy) in terms of magnifying power, field of view, focal distance, intensity, color of light and costs. Brightness provided by the instruments was measured with a professional exposimeter (Bowens flash meter III, Sekonic Electronics Inc, Japan) and converted in lux in order to account for the distance between light source and target.

**Fig. 4:**
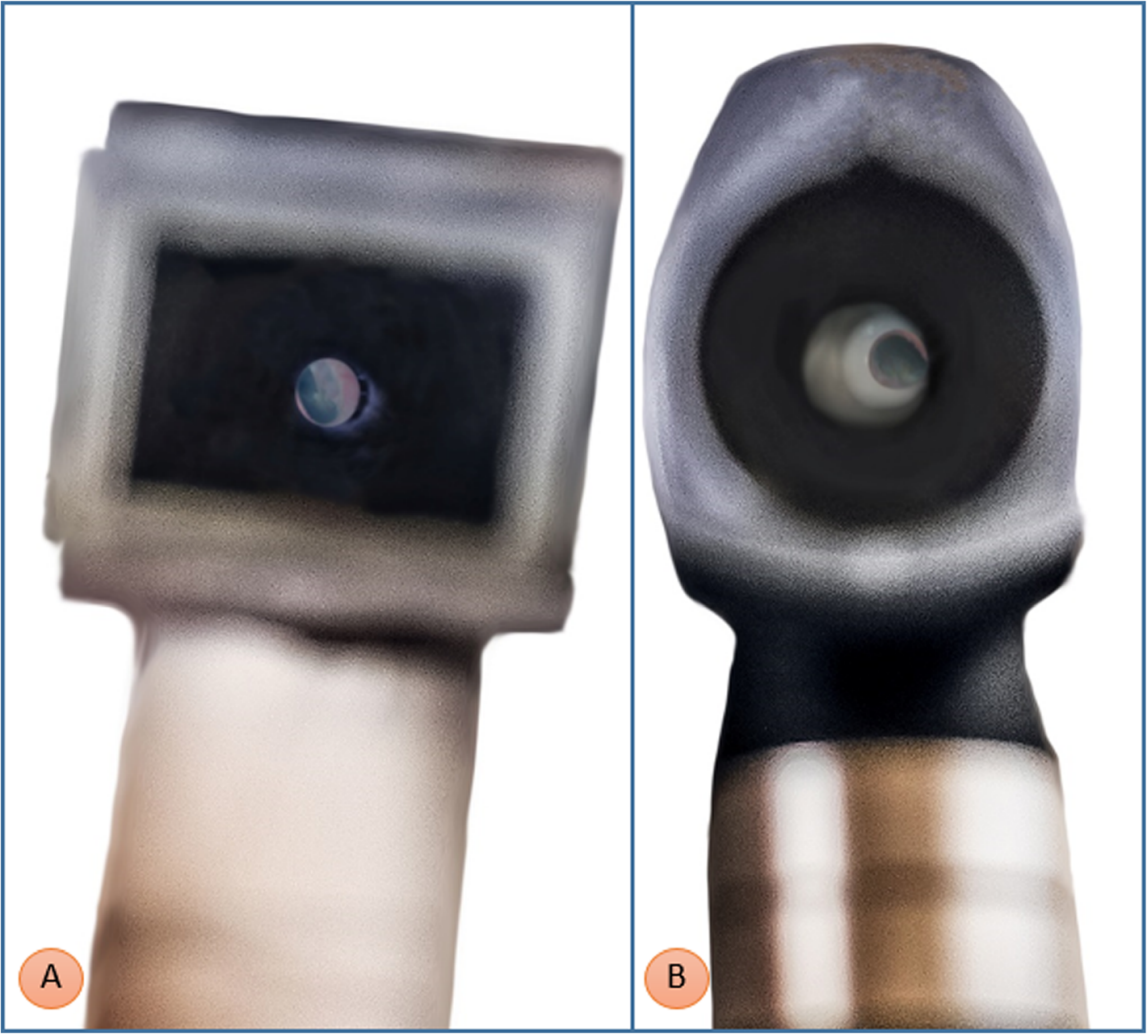
Comparison of otoscopes on volunteers showed encouraging preliminary results

**Fig. 5:**
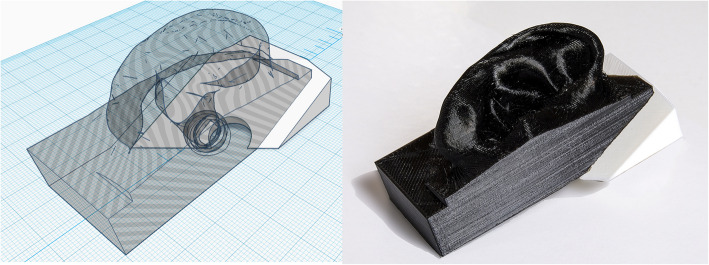
3D representation and picture of the model of the external ear used for otoscope testing

Initial testing on us as volunteers showed images as clear as seen through the commercial solution (Fig. 4). In order to properly test the devices, we developed an evaluation model using an imaging quality millimeter scale, designed for comparing photographic lenses [19]. Then, to recreate the lightning conditions of the tympanic membrane, we adapted a 3D model derived from actual CT-scans of the external ear [20]. The object was cut in two parts by a plane passing through the tympanic membrane. In this way, the imaging quality scale was positioned exactly in place of the eardrum at the inner end of the ear canal. The external part, which comprises the ear lobe, was printed using flexible TPU at 10 % infill, in order to mimic elasticity of ear tissue and to allow the otoscope tip to fully insert into the ear canal. This recreated the diagnostic maneuvers performed in usual clinical practice. The ear lobe is total black, thus reducing light refraction. The ear was put atop a base printed using white ABS, forming a fixed support for the millimeter scale (Fig. 5).

## Results

**Fig. 6:**
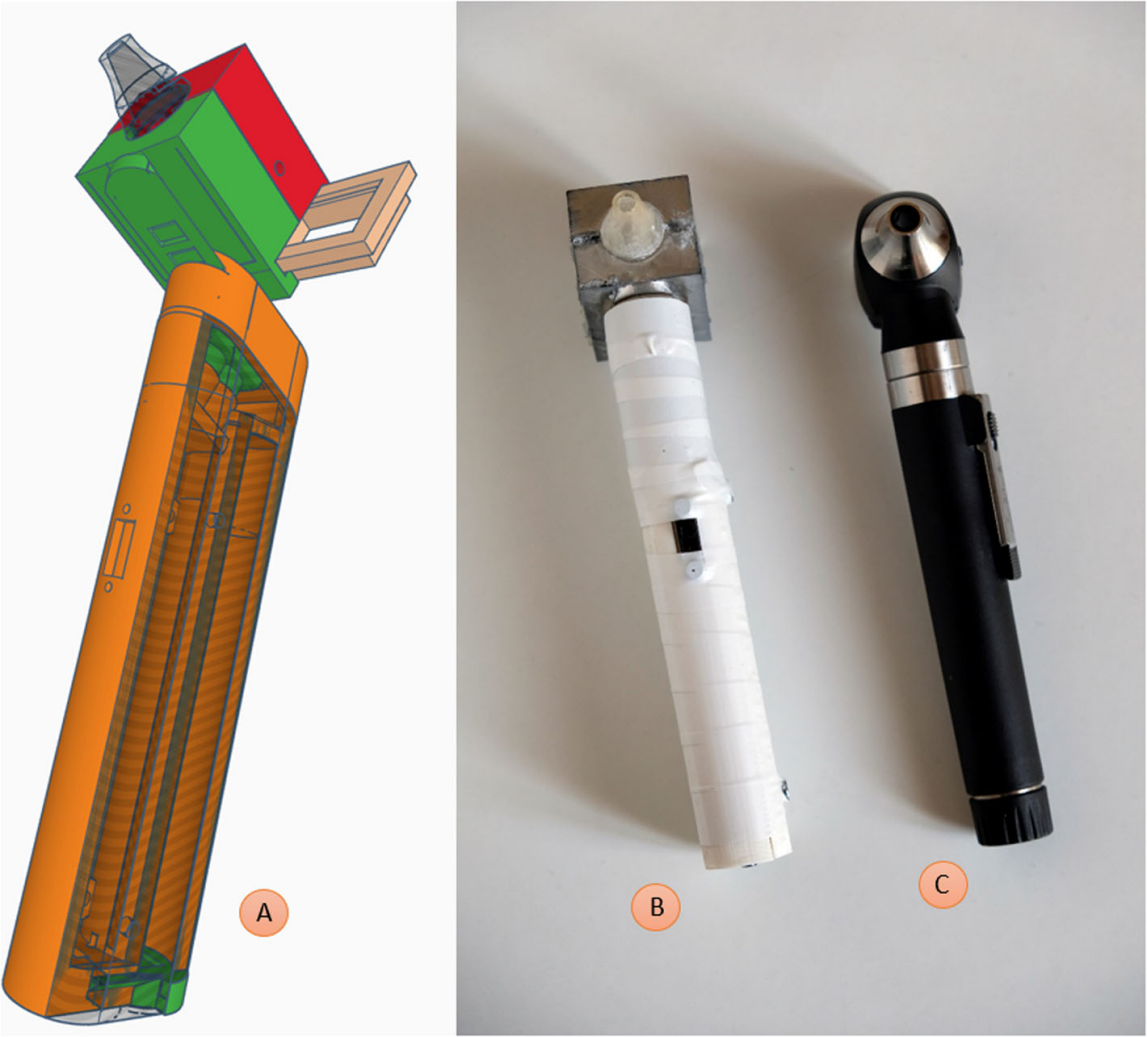
3D model of the prototype with interchangeable heads and removable lenses, and visual comparison with a commercial otoscope

In Fig. 6 the otoscope is shown as a 3D rendering and as a picture. 3D final models - head and handle - of the otoscope are freely available at https://archive.org/details/otoscope_20210921 in printer-friendly .stl format. Full building instructions are reported in Appendix 1.

### Comparison of otoscopes

Results of the comparison of otoscopes are reported in Table 1.

**Table 1:** Technical features of the prototypes versus a commercial solution (Sigma F.O. LED, G.I.M.A. S.p.A., Milan, Italy)

Otoscope	Magnifying factor	Field of view (mm)	Focal distance (cm)	Brightness (lux)	Color temperature (kelvin)	Cost
- White light, self-fabricated	3x (6x with a second lens)	4	0-2	70	6700	< 5 Euros
- UV, self-fabricated	3x (6x with a second lens)	4	0-2	7	n.a.	< 5 Euros
- Gima	3x	4	0-3	40	5000	Around 100 Euros

The magnifying factor of our self-fabricated otoscope was determined in 3x power, which was comparable to the commercial otoscope. However, our design includes the possibility of implementing a second Fresnel lens in the central part of the otoscope head. This could increase magnification power up to 6x, depending on the power of the lens chosen. In other dimensions we registered a substantial overlap between the two devices, while the commercial solution has a better ability to focus on distant objects. Since the length of the ear duct is about 2.5 cm and the tip of the head of the otoscope is inserted in the ear, the capability to focus at 2 cm from the tip should provide a clear vision of the eardrum. UV light was seemingly less intense than white light. This was expected since the amount of visible light provided by UV LEDs is limited. When a fluorescent material is illuminated, however, the amount of colored light reflected is sufficient for an easy visualization of the target. Color temperature between the white-light prototype and the medical device resulted similar, since both values are included in the daylight lightning spectrum and are commonly used in medical diagnostic tools. The costs of the two devices differed by more than tenfold, the self-fabricated otoscope with one Fresnel lens costing about 5 Euros. In Appendix 2 we provide a detailed reporting of costs associated with self-fabrication of an otoscope.

### Quality of vision

**Fig. 7:**
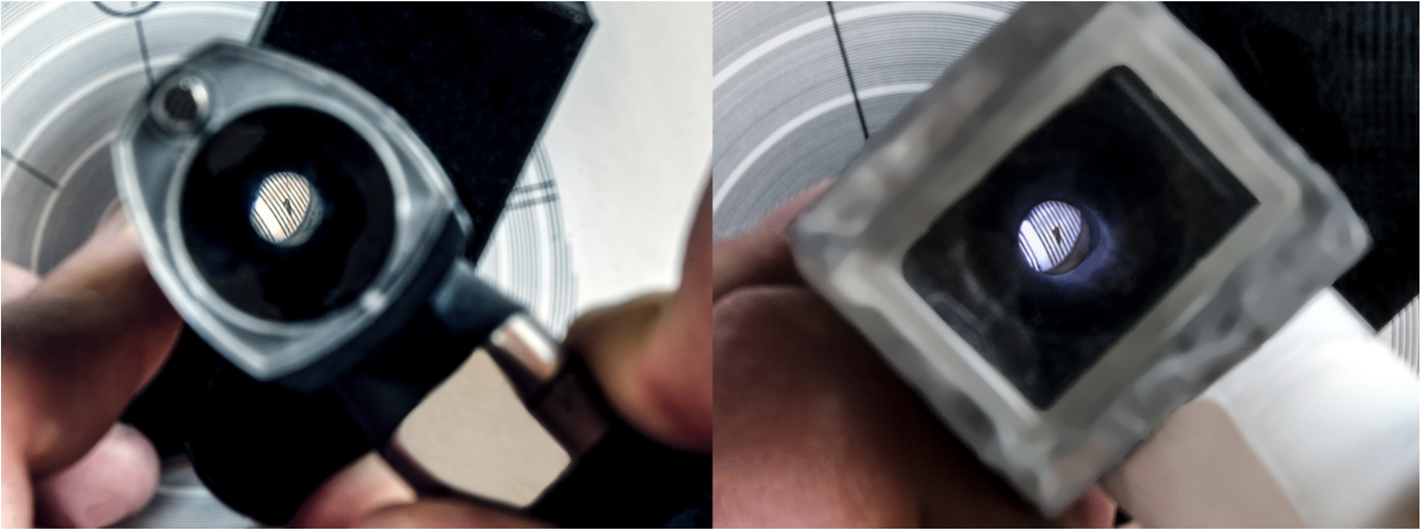
Comparison using a photographic lens quality scale (right: prototype; left: Gima otoscope)

Both otoscopes were able to clearly see the gaps between the smallest test lines, which are placed at a 0.2 mm distance. This result demonstrates similar overall quality between the instruments, largely sufficient to clearly see the malleus and the vast majority of lesions of the tympanic membrane (Fig. 7).

### Safety

Devices used in clinical practice must meet precise safety criteria. Employed materials should be washable and antibacterial. FDM 3D prints are not impermeable nor sterilizable. However, the otoscope tip is not directly at contact with the patient’s skin. The prototype makes use of certified, disposable specula as usual, which are fixed in a specific location of the tip. We also used a washable tape on the handle to improve grip and to allow easy decontamination. Circuits should be certified and waterproof to limit risks (e.g., short circuit). Otoscopes use a low voltage (3 V) which is reassuring against major electric shocks. For all these reasons, we can assume that our 3D printed otoscope is safe for medical use. However, the current European Medical Device Regulations requires both devices and their production process to reach the end of a complex certification procedure before being sold or used in clinical practice. The only exceptions to this rule are scientific trials or emergency use, which match our intended scenario.

## Discussion

Otoscopy is an important diagnostic procedure. The main diagnoses associated with its use are cerumen impaction, acute otitis media, and movements and lesions of the tympanic membrane. These problems are frequently seen in general practice and pediatrics. Severe complications of otitis media, including intracranial abscesses, meningitis and sinus thrombosis, are rare but early diagnosis is necessary in optimizing prognosis [21].

Our key assumption moving us in developing a low-cost otoscope is that quality otoscopes are not as frequent as they should be in community and hospital settings. One potential barrier might be the relatively high cost of these devices, particularly in low-resource settings where affordability is a key driver. Although some cheap otoscopes are available for purchase in e-commerce sites, these devices might be inadequate for clinical use. Most of the <50€ instruments do not use removable lenses, and do not provide information on lightning quality or temperature of light. These features are typical of medical grade otoscopes, and are essential to ensure a clear vision and to allow medical procedures, such as foreign body removal and pneumatic examination. When trying to design the most inexpensive otoscopes, early developers initially took inspiration from these devices [22]. Resulting prototypes might be appropriate as starting point, however improvements are needed before their introduction in clinical practice. As part of the response to the 2015 Nepalese earthquake, consumer-maker communities supplied 3D printed otoscopes to health posts in an attempt to support local health professionals. The final design, after having gathered feedback from a range of medical practitioners in the UK and Nepal, was published in 2017 [10]. Our self-fabricated otoscope is built by drawing inspiration from the Nepalese earthquake experience, refining several development elements. We tested it to ensure that medical practitioners can use it to cure with confidence. Its quality resulted comparable to expensive medical grade products and it should be considered as a micro-scale production serving single clinicians or underserved communities, in those settings where local regulations allow use of self-made devices.

When compared with certified products, self-fabricated otoscopes have important limitations. Performance does not depend entirely on the quality of design, but also on the practical skills of those in charge of the assembly. When building our otoscope, we observed that even a millimetric difference in LED positioning resulted in a noticeable change in light focus, which translated during tests in a consistently darker or brighter eardrum. Therefore, it is important to check every assembly directly with final users, in order to adapt the products to the clinicians’ needs. Registered medical devices have to meet strict safety criteria to be approved for clinical use. The Maker’s approach to technology does not follow industrial standards, which may constitute an insurmountable barrier when trying to reach a certification of the production process. However, quality of vision of our otoscope was comparable to the performance of a high-standard product, and safety issues can be also ruled out due to the intrinsic characteristics of this kind of instruments, designed for external use. The device seems to be reliable over a long period of operation as it has a solid build quality in terms of the materials used. However, we lack long-term empiric usage data. The materials and components included in our design are commonly available. Purchasing these components should not represent a problem even during health crises, unless the crisis is so long to cause a supply chain disruption. The Fresnel lens is the most difficult part to obtain. However, any available lens could perform the same task, if it fits in the lens holder piece. Another possibility is to use the attached .stl file for directly printing a lens using transparent resin, a time-consuming work.

The consumer-maker community has an important role also in exploring improvements of devices. We were able to rapidly design and develop an UV otoscope. These devices are scarcely available in the market. Mycotic external otitis is a recurrent problem in general practice. Therapy is empirical and prone to mistakes, due to clinical features overlapping with bacterial external otitis. A diagnosis currently requires microscopical examination of the fungal material. Excluding an otomycosis will also limit the use of anti-fungal therapies, decreasing risk of resistance. This simple instrument could provide a faster and direct diagnosis, supporting better treatment decision-making.

## Conclusions

Low access to key diagnostic tools is detrimental to priority health needs of populations. Ear pain, tenderness, and different degree of hearing loss are common problems which require otoscopy as first diagnostic assessment to be considered. Where an otoscope is not available because of budget constraints, a 3D printed, low-cost, otoscope might represent a feasible opportunity. There is the need for more open access medical device projects, which should be available and validated in prevision of future shortages and emergency situations. Further studies which encompass a cooperation between Makers and health care institutions for the development and scientifical testing of well-established devices should be encouraged.

## Supplementary information

**Additional file 1**

**Additional file 2**

## Data Availability

All data, building instructions, and 3D printer files for this study are open source and can be downloaded for free from https://archive.org/details/otoscope_20210921.
