# Supplementary material for: An open-access and inexpensive 3D printed otoscope for low-resource settings and health crises
Source: 3D Print Med. 2021 Nov 17;7:36. doi: 10.1186/s41205-021-00127-3 (PMC8595962; doi:10.1186/s41205-021-00127-3)
Supplement: Supplementary file 1 — Additional file 1 [file 41205_2021_127_MOESM1_ESM.docx]

# Otoscope building instructions

In the following pages we report the full building instructions for an affordable otoscope with interchanging heads. Only common use materials and electronic are required. It is easy to build and it should provide medical-grade performances when correctly assembled.

All needed files, including .stl for 3D printing, can be downloaded from here: <https://archive.org/details/otoscope_20210921>

***List of materials***

- 2 M3 15 mm screws (side of the handle)
- 3 M2 6 mm screws (switch and battery compartment)
- Two springs from ball-pens
- 6 white 5mm, 3.3V LEDs (for the white light otoscope) or
- 6 5mm, 3.3V UV LEDs (for the UV otoscope)
- A 3-pin switch (<https://www.amazon.it/gp/product/B06XGTKK9Z/>)
- Nickel-plated strip tape (<https://www.amazon.it/gp/product/B07MM88TJZ/> )
- 28 AWG Electric wiring (<https://www.amazon.it/gp/product/B07PN8GD6G/>) or similar
- Fresnel lens, magnification factor 3x, 1mm thickness. The other measures do not matter as it can be cut into the needed shape (<https://www.amazon.it/Ingrandimento-Formato-Magnifier-Raccoglitore-Firestarter/dp/B06W5FCS4Q/>)

***Assembling the handle***

3D print all parts, orienting them appropriately. Remove the support structures at the end of printing. For these prints, we recommend a FDM printer; when a PLA filament is chosen, we suggest to set 200 °C for the extruder and 60 °C for the printing surface; when using ABS, we set 240 °C for the extruder and 100 °C for the surface.

When having access to a resin printer, the top part can benefit from the higher resolution of SLA printing. Using resin printing for the piston and this component, which includes the lodging for the piston, allows more precise movements and reduces the need to file the printed parts.


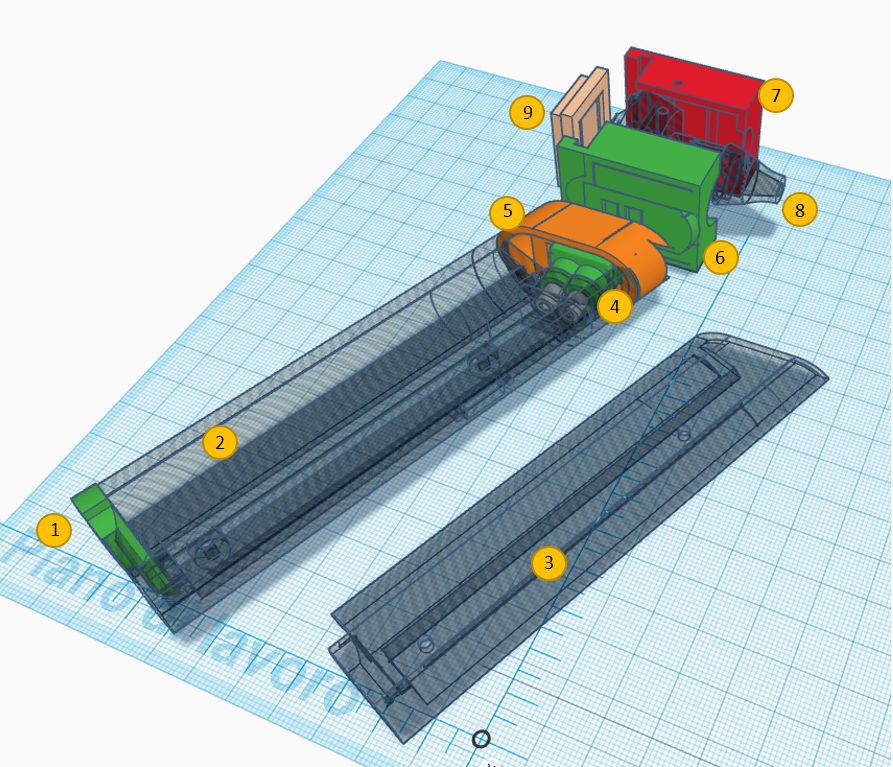


| **N** | **Part** |
| --- | --- |
| 1 | Battery compartment |
| 2 | Lower handle |
| 3 | Handle cover |
| 4 | Piston |
| 5 | Upper handle |
| 6 | Lower head |
| 7 | Upper head |
| 8 | Lightning ring |
| 9 | Lens holder |

Fig 1. Part list and positioning.

After completion of the prints, glue some length of nickel strip to the covering of the battery compartment, filling the notch and the internal edge. Use a second strip to coat the inside of the hollow, especially the deepest part. Solder a wire to the strip inside the handle, then solder the other extremity to the closest pin of the switch.


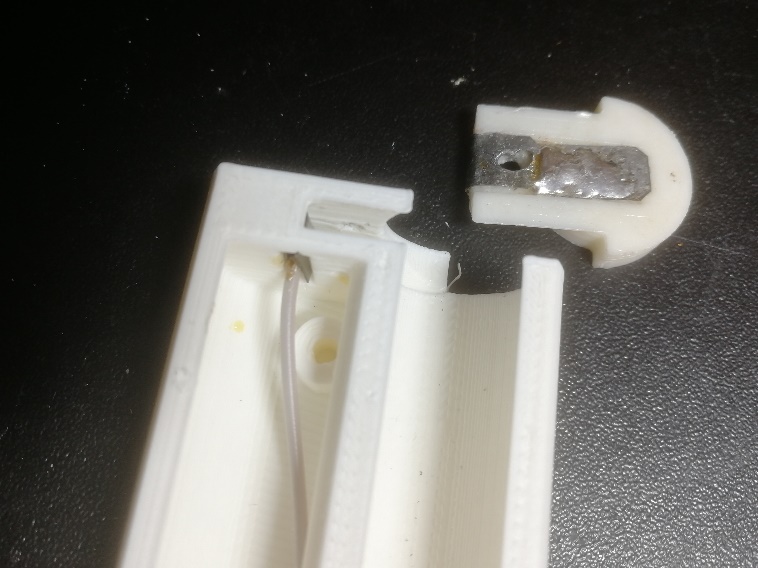


Figure 2. Wire connection to the battery compartment.

Solder another wire to the central pin. This wire will go through the upper opening and through the hole on the right side of the piston, going through it and then exiting from the upper-side hole. There is a hole in the upper part of the battery compartment. Put a spring through it. The length of the spring on each side can be reduced or increased to tweak the tension on the batteries. Solder a wire to the upper part of the spring.


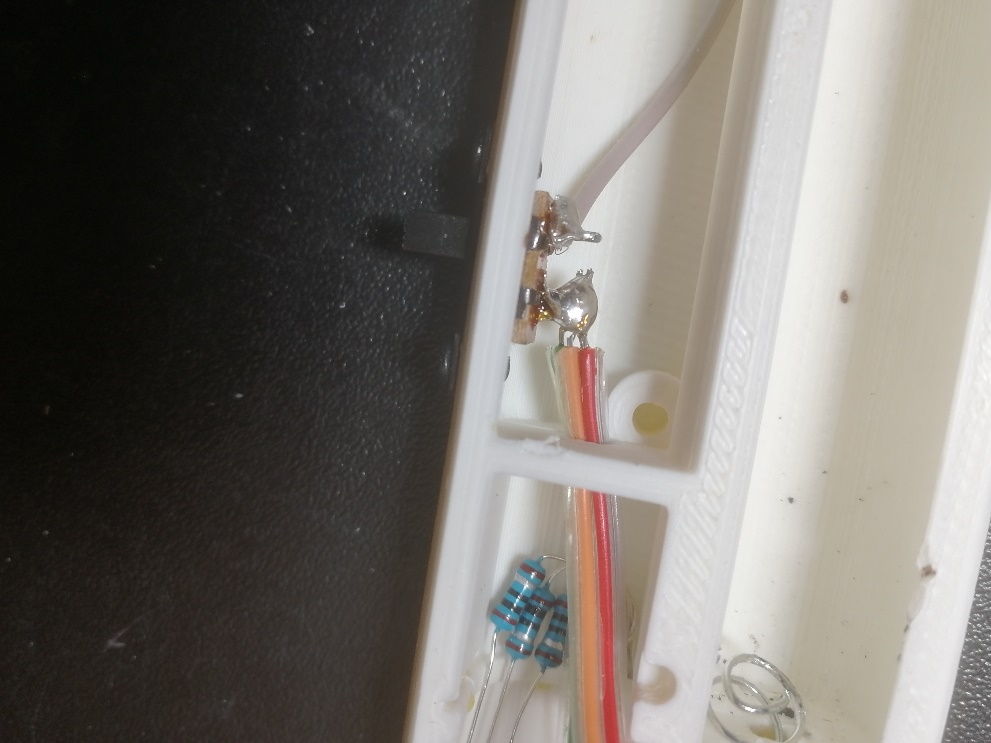


Figure 3. The switch and upper battery compartment. Optional resistors (220 Ohm) can be interposed between each LED anode and the switch.

Run this wire through the left side opening on the piston, then it should exit from the upper hole. Cut a ball-pen spring in half and use the 2 halves to fill the piston’s lodging. Close the handle using the M3 screws, then glue (it is best to use epoxy) the top part of the handle to the main part. Double check that no glue blocks the movement of the piston. Eventually, use lubricant to smooth the movement.

Solder 2 small pieces of nickel strip tape to the wires exiting the piston. The heat of the soldering iron should be sufficient to slightly melt the plastic, gluing the strips to the piston.


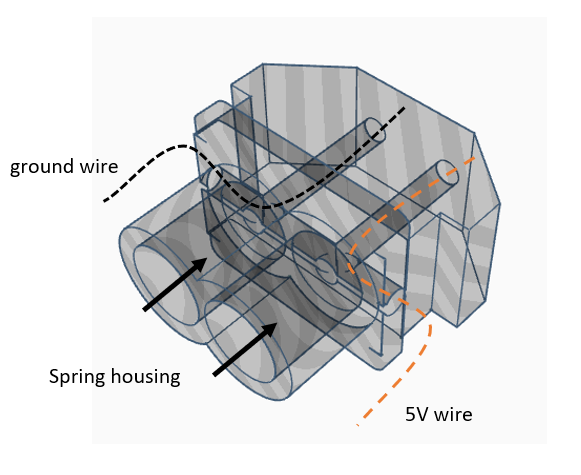


Figure 4. Preparing the piston.

***Assembling the otoscope head***

The central piece has to be printed using transparent resin or filament. We recommend using transparent resin for better results. The other parts are best printed with opaque filament/resin.

Place the LEDs in their lodgings in the central piece. Use white LEDs for a traditional otoscope, UV LEDs for a fluorescence device. Glue them into position (we used a glue gun for this task). Connect all positive LED PINs and solder them. Repeat with all negative PINs. Solder one wire to the positive ring and one to the negative one. Put a small piece of the nickel strip in each of the two openings in the inferior piece, ensuring that the inferior part is smooth and has no gaps. Then, for each opening, solder together the two extremities of the nickel strip. Solder the positive wire to the anterior opening strip, the negative one to the posterior strip.


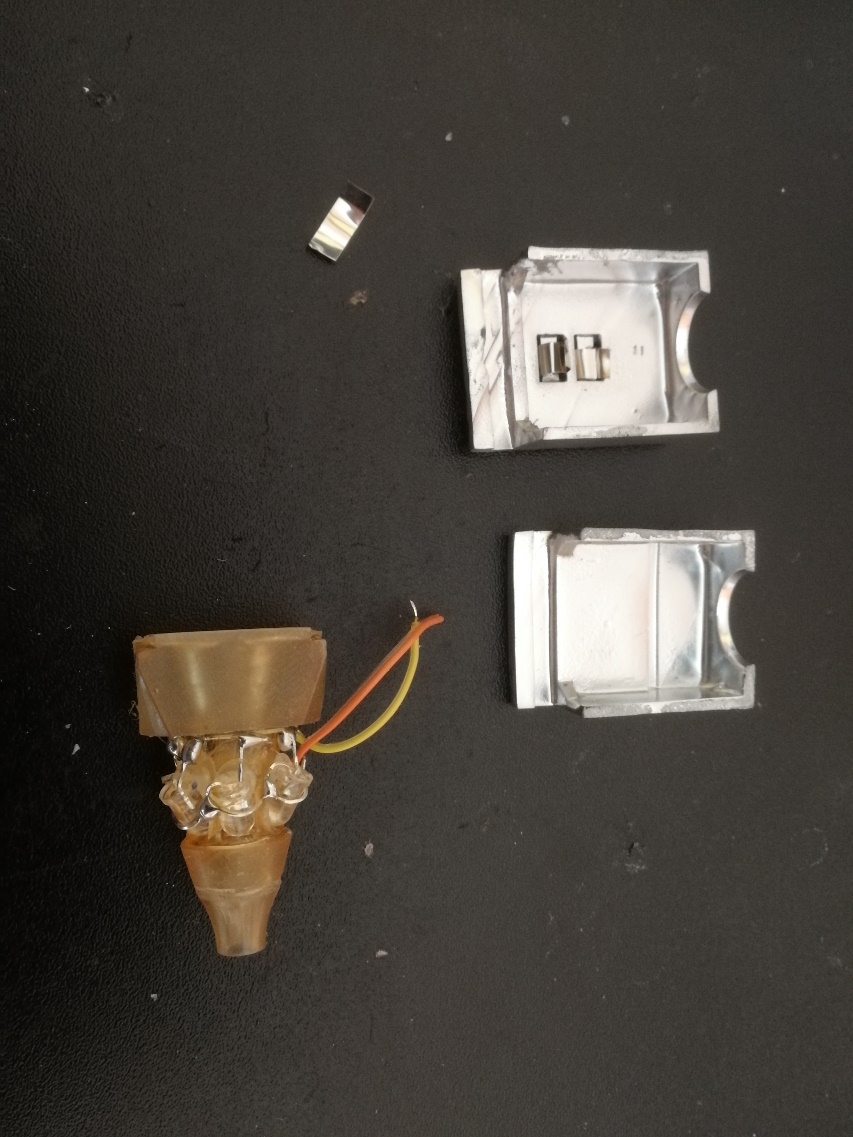


Figure 5. LED positioning and connection in the head of the otoscope.

Paint the inside of the central piece with dark paint, to avoid light reflections from the LEDs. Glue together the upper, central and lower piece. Cut the central part of the Fresnel lens to a measure small enough to slide it in the opening of the lens holder. Slide the lens holder in position through the side of the head. Glue the lens holder to the otoscope’s head. Optionally, cut the central part of a second Fresnel lens and slide it through the opening on the top side of the head. Use hot glue to seal the entrance.


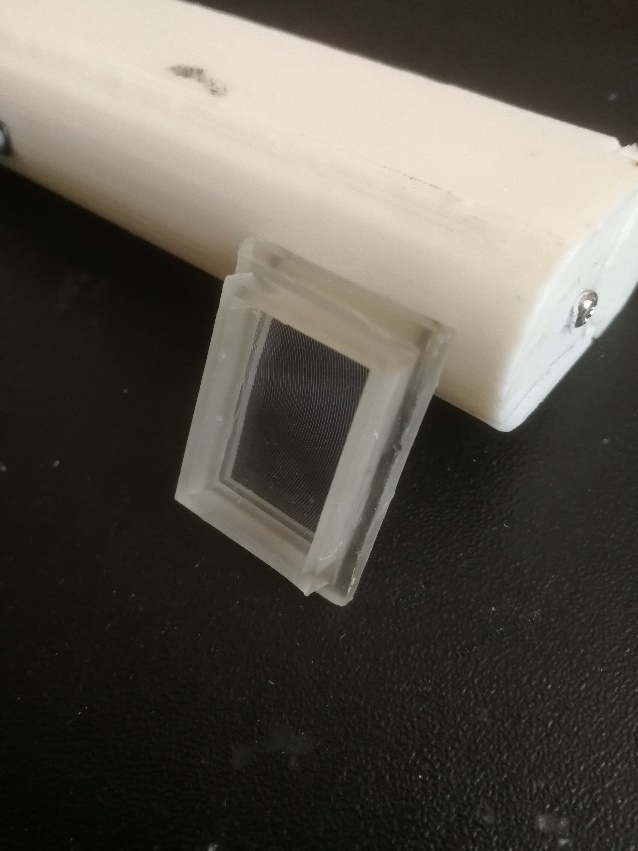


Figure 6. Insert the Fresnel lens into the lens holder.
