# Supplementary material for: An open-access and inexpensive 3D printed otoscope for low-resource settings and health crises
Source: 3D Print Med. 2021 Nov 17;7:36. doi: 10.1186/s41205-021-00127-3 (PMC8595962; doi:10.1186/s41205-021-00127-3)
Supplement: Supplementary file 2 — Additional file 2 [file 41205_2021_127_MOESM2_ESM.docx]

**Manufacturing cost details**

The cost of our devices was 20 times smaller in comparison with entry level professional ones, which are sold around 100 Euros. Costs for materials include electronic components and for 3D printing (Table 1). Resin prints are more expensive than ABS or PLA prints. Currently, a bottle of 1 liter of standard clear resin can be bought in e-commerce websites at 30€/L, while 1 kg of ABS filament costs around 15€. On the opposite, FDM printing technique consumes more energy for the heating of nozzle and bed. We considered a mean power consumption of 0.05 kWh for filament printing, 0.03 kWh for SLA [1]. We reported in the table only the costs for raw materials. Time consumption may also be an important factor. Each part of the first prototype initially took us 3 hours to assemble. While the process was noticeably expedited in the following iterations, it still remains a significant, difficult to estimate, and often overlooked, cost. However, we noticed that once we took confidence with the building process, we were capable of soldering and assembling a full otoscope in about 30 minutes. Costs listed in the table do not include initial expenses for buying a 3D printer, which could range from 200$ to several thousands, and for maintaining hardware or training staff. Therefore, this comparison is accurate when a potential user already owns a 3d-printing laboratory.

| **Manifactured part** | **Cost (€)** |
| --- | --- |
|  |  |
| ***Handle*** |  |
| - 3D printed parts SLA (FDM) | 1.28 (0.64) |
| - SLA electricity consumption (FDM) | 0.07 (0.12) |
| - 7 cm nickel strip | 0.04 |
| - Switch | 0.09 |
| - 16 cm wire | 0.03 |
| - Screws | 0.06 |
| **Total SLA (FDM)** | **1.57 (0.98)** |
|  |  |
| ***Otoscope Head*** |  |
| - 3D printed parts SLA (FDM) | 0.55 (0.28) |
| - SLA electricity consumption (FDM) | 0.05 (0.09) |
| - 6 LEDs (or 6 UV Leds) | 0.3 (0.42) |
| - 8 cm wire | 0.02 |
| - 3 cm nickel strip | 0.02 |
| - Fresnel lens | 1.9 |
| **Total SLA (FDM)** | **2.84 (2.61)** |

Table 1. Manufacturing cost details.

**References**

1. Walls S, Corney JR, Vasantha GVA (2014) Relative energy consumption of low-cost 3d printers. Proceedings of the 12th International Conference on Manufacturing Research, Southampton, UK, January 2014.
